# Supplementary material for: Neuro-genetic plasticity of Caenorhabditis elegans behavioral thermal tolerance
Source: BMC Neurosci. 2019 Jun 10;20:26. doi: 10.1186/s12868-019-0510-z (PMC6558720; doi:10.1186/s12868-019-0510-z)
Supplement: Supplementary file 1 — Additional file 1. Supplementary figures. [file 12868_2019_510_MOESM1_ESM.docx]

Supplementary figures


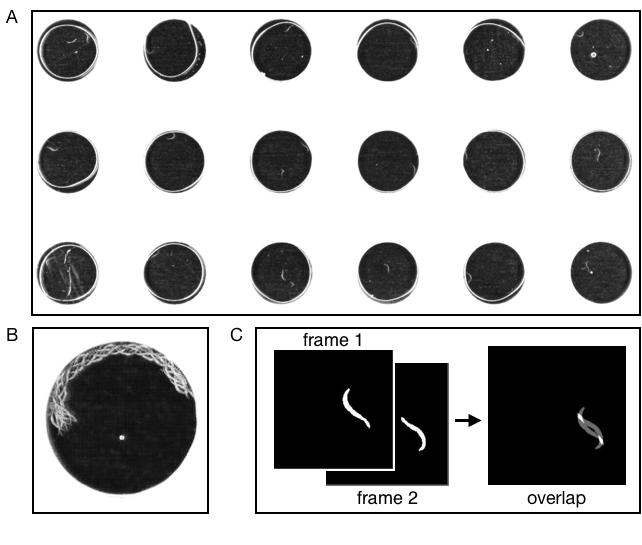


Supplementary Figure S1. Droplet data and processing. (A) Raw image of micro-droplet array. Each droplet contains NGM buffer and a single worm. (B) A swimming worm from a single droplet shown as a 20 sec multiple exposure. (C) Movement of worms is quantified as the proportion of overlapping worm images separated in time. Two frames are binarized to segment the worm image and then the non-overlapping fraction of the images are calculated. The locomotion index is calculated as the fraction of non-overlapping pixels (grey) divided by the total pixels for both images (white and grey).


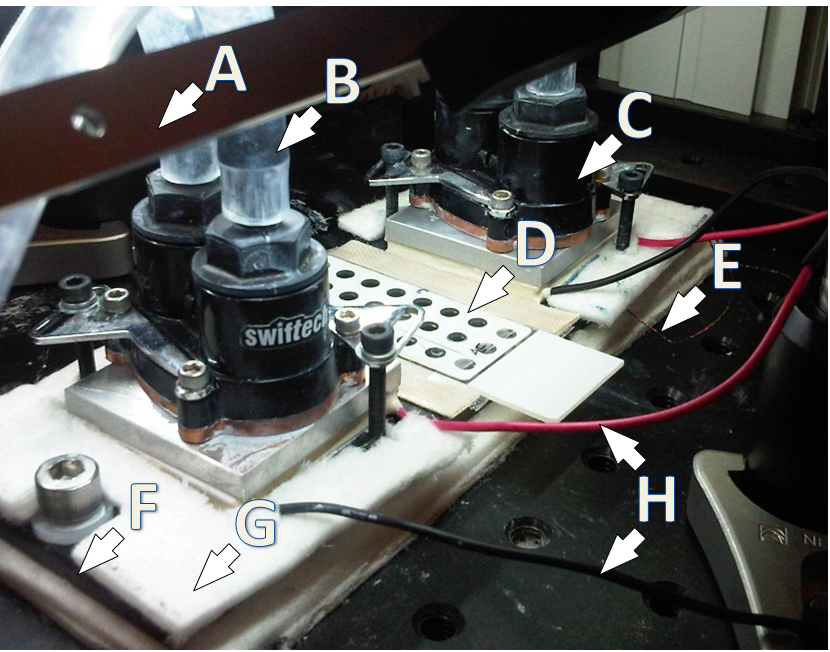


Supplementary Figure S2. Droplet apparatus and setup. (A) LED obliquely illuminating light strip (B) water tube to temperature-controlled water bath (C) water block connected above thermo-electric cooler (TEC) pad (D) sample slide with droplets and cover slip (E) thermocouple wires for detecting temperature of aluminum block (F) edge of aluminum block/stage (G) fiberglass insulation around aluminum block (H) wires from TECs to I/O controller. Not pictured are the I/O controller and the camera mounted 24 cm above the stage. For scale, each circular well at (D) is 4mm in diameter and TECs are 45mm apart.

Supplementary Figure S3. Locomotion Index. (A) Locomotion Index at constant temperatures. LI1 is nearly constant for the duration of the experiment (21 min) at benign temperatures. (B) Longer term (58 steps ~ 77 min) effects of brief high-temperature exposure. High temperature exposure of 80 sec occurs at dotted line. Control worms held at a constant 23°C (black line) for the whole experiment gradually slowed their locomotion to approximately 60% of the level seen at the start of the experiment. This slowing of average locomotion occurs because a fraction of worms stop or entering cycles of episodic swimming rather than from all individuals slowing down. *n*=18 worms tested for each condition, minimum of 8 worms included in means. Error bars are ± SEM.

Supplementary Figure S4. (A) Thermal reaction norms of swimming behavior for 58 *C. elegans* genetic mutant strains (gray and red lines) and two wildtype strains (black line = N2, dashed line = CB4856). All worms reared at 23°C; 17 - 36 individuals included in calculations at each assay step for each strain (Table 1). TTCs for subsets of mutant strains with sensory disruptions from (A) are shown for thermosensory defects (B), chemosensory defects (C), and mechanosensory defects (D). (E) TTCs for strains with gene mutants affecting dopamine signaling. (F) TTCs for strains with mutations in genes involved in heat shock response, oxygen sensing, and muscle activity. Error bars in (B-F) indicate ± SEM; TTCs for wildtype strains N2 and CB4856 are shown in all of (A-F) for reference.


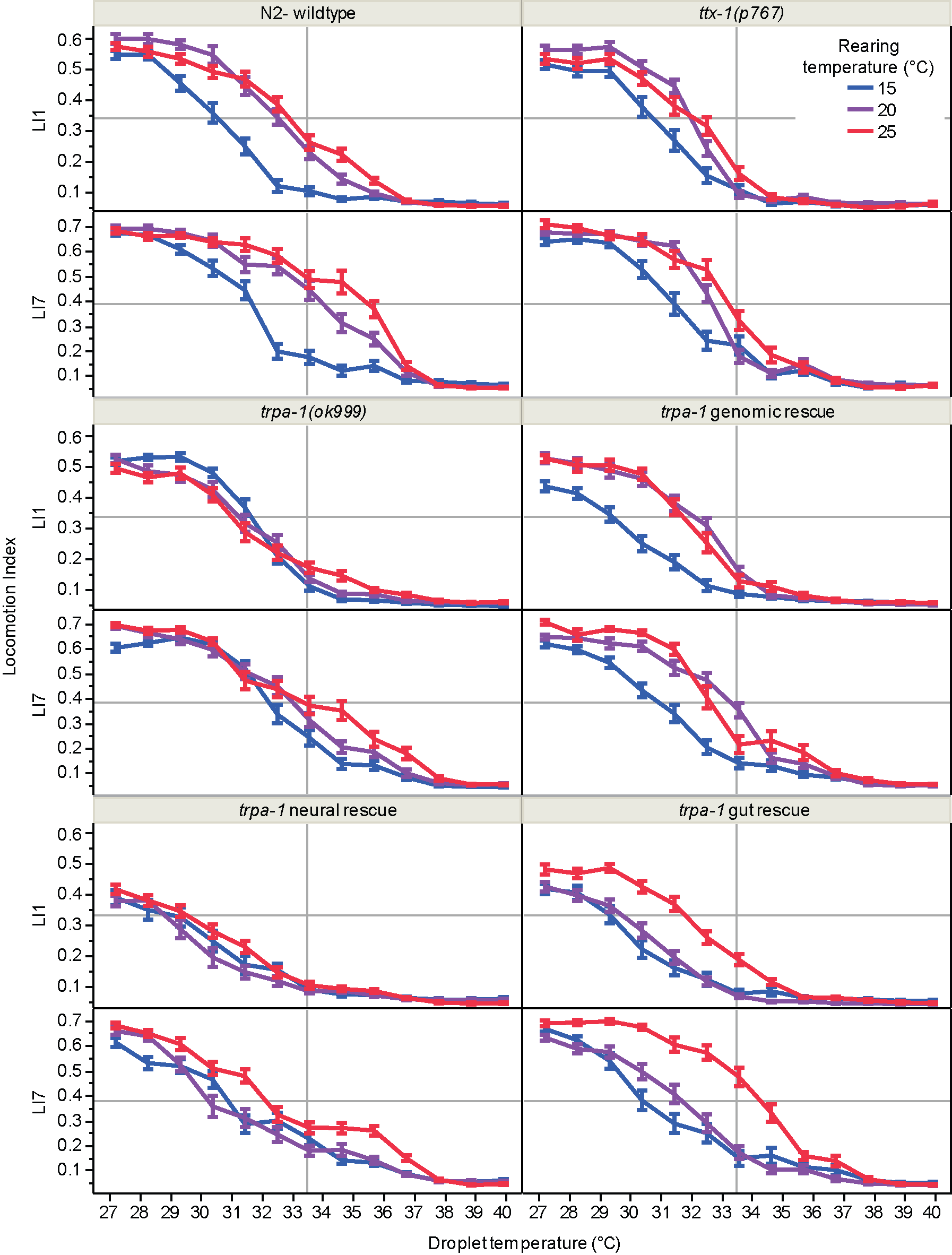


Supplementary Figure S5. Plasticity of thermal performance response to rearing temperature of tissue specific *trpa-1* rescues.


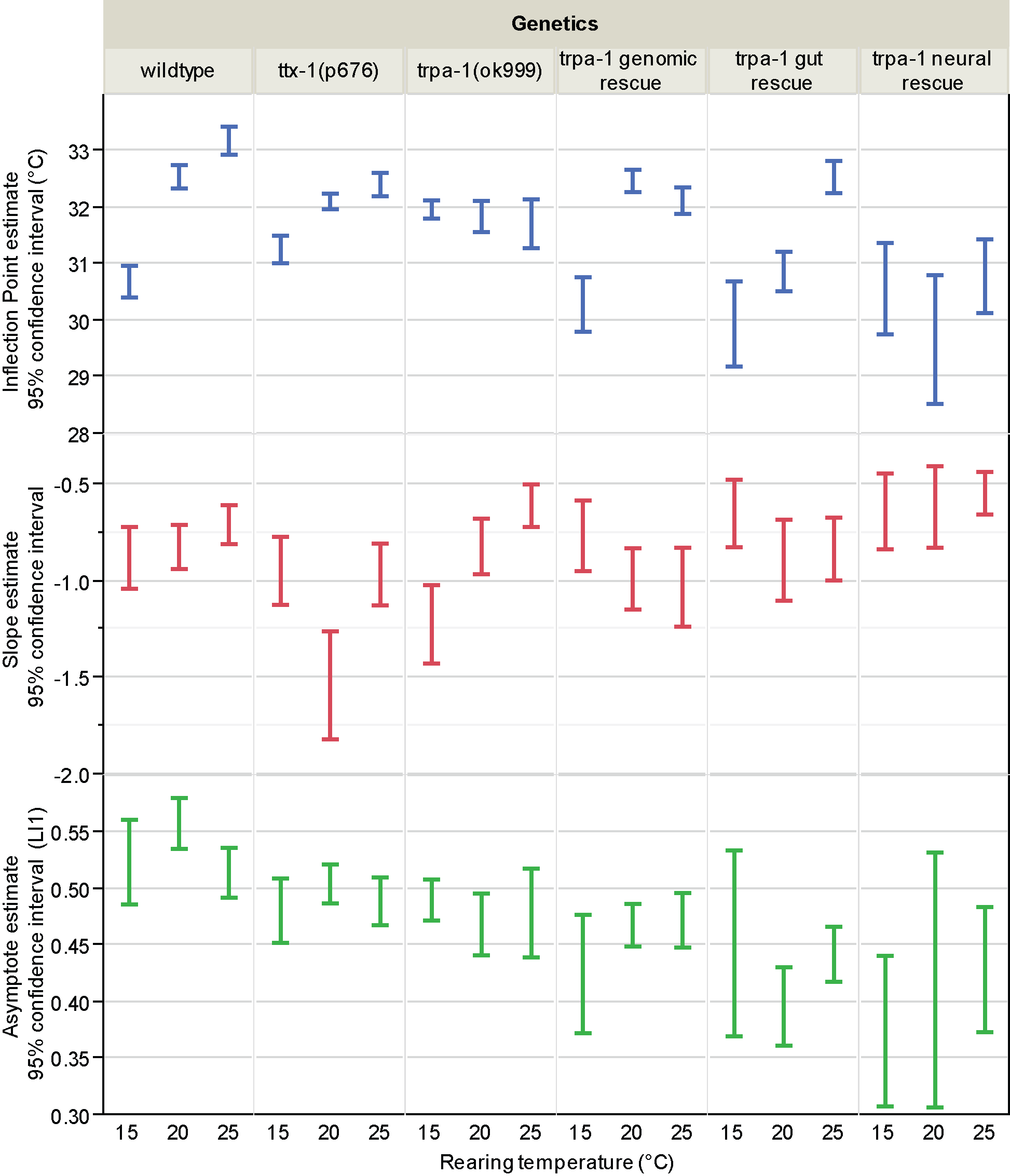


Supplementary Figure S6. Function fit parameter estimate 95% confidence intervals for *trpa-1* rescue experiment. 95% confidence intervals for three parameter logarithmic function fit to LI1 data for strains in *trpa-1* rescue experiments at three rearing temperatures.
